# De novo truncating variants of *TRIM8* and atypical neuro-renal syndrome: a case report and literature review

**DOI:** 10.1186/s13052-023-01453-4

**Published:** 2023-04-15

**Authors:** Wei Li, Hui Guo

**Affiliations:** 1 Department of Child Health Care, West China Second University Hospital, Sichuan University, No. 20, Section 3, Renmin South Road, Wuhou District, Chengdu, 610044 Sichuan China; 2 Key Laboratory of Birth Defects and Related Diseases of Women and Children (Sichuan University), Ministry of Education, Chengdu, Sichuan 610044 China; 3 Department of Pediatric Nephrology, West China Second University Hospital, Sichuan University, No. 20, Section 3, Renmin South Road, Wuhou District, Chengdu, 610044 Sichuan China

**Keywords:** *TRIM8*, Focal segmental glomerulosclerosis, Proteinuria, Epilepsy, Case report

## Abstract

**Background:**

The *TRIM8* gene encodes a protein that participates in various biological processes. *TRIM8* variants can lead to early termination of protein translation, which can cause a rare disease called neuro-renal syndrome. This syndrome is characterized by epilepsy, psychomotor retardation, and focal segmental glomerulosclerosis. However, we found that some patients may not present the above typical triad, and the reason may be related to their variant sites.

**Case presentation:**

We report a case of a 6-year-old boy with nephrotic-range proteinuria as the first prominent manifestation of *TRIM8* variant. He had stage 3 chronic kidney disease at the time of presentation, specific facial features, and a neurogenic bladder. He had not experienced seizures previously. There were no apparent abnormalities in his growth, intelligence, or motor development. The results of whole exome sequencing showed a *TRIM8* variant. Renal biopsy revealed focal segmental glomerulosclerosis and renal tubular cystic dilatation. He did not respond to hormone and angiotensin-converting enzyme inhibitor treatment; however, the symptoms of neurogenic bladder were relieved after treatment with Solifenacin.

**Conclusion:**

In this case, renal disease was the prominent manifestation; the patient had no other obvious neurological symptoms except a neurogenic bladder. Notably, the variant site is the closest to the C-terminal to date. Based on the analysis of previously reported cases, we found that as the *TRIM8* variant became closer to the C-terminal, the renal lesions became more prominent, and there were fewer neurologic lesions. Our findings provide a new understanding of neuro-renal syndrome caused by *TRIM8* variant. Patients may only have kidney disease as a prominent manifestation. At the same time, we found that we should also pay attention to the eye lesions of these patients. Therefore, gene analysis is helpful in identifying the etiology and guiding the prognosis of patients with hormone-resistant proteinuria. We suggest that *TRIM8* should be included in gene panels designed for the genetic evaluation of hormone-resistant proteinuria.

**Supplementary Information:**

The online version contains supplementary material available at 10.1186/s13052-023-01453-4.

## Background

The *TRIM8* gene is located on human chromosome 10 and encodes a protein with 551 amino acids. The protein is an E3 ubiquitin ligase that participates in biological processes such as cell signaling, proliferation, differentiation, autophagy, immunity, and tumor growth. Its dysfunction is closely related to cancer, inflammation, and autoimmune diseases [1, 2]. *TRIM8* is widely expressed in human tissues, especially in the central nervous system, kidney, and eyes [3]. Therefore, variants in the *TRIM8* may damage the nervous and renal systems. Eight reports have described neurological and renal system diseases associated with *TRIM8* variants, and all of them were de novo truncating variants in the last exon of *TRIM8* [4–11]. A total of 22 cases have been reported in the literature, and all of them were observed in children. Among them, three cases involved only neurological diseases (mainly epilepsy with psychomotor retardation), two cases involved renal diseases only, and two cases involved renal diseases accompanied by mild psychomotor retardation (no seizures). The other 15 cases involved obvious symptoms in the neurological and renal systems. The main manifestations of renal lesions are nephrotic-range proteinuria, and most cases eventually progress to end-stage renal disease. Renal biopsy revealed focal segmental glomerulosclerosis (FSGS), and only one case involved FSGS with renal tubular damage. Most reported cases included specific facial features (such as long philtrum, straight eyebrows, sunken eyes, microcephaly, micrognathia, broad forehead, broad nasal bridge, upper-slanted palpebral fissures, thin lips, large ear lobes, and low-set ears). This study presents a case of one boy who had a de novo truncating variation in the last exon of *TRIM8*. Notably, this type of variant has not been reported before. Of all the previously reported variants, the variant involved in this case was the closest to the C-terminal. We reviewed the characteristics of previously reported cases of neuro-renal syndrome caused by a *TRIM8* variant and found that when the *TRIM8* variant is closer to the C-terminal, the renal damage is more severe and the nervous system damage is less severe [4–11]. Our findings provide a new understanding of neuro-renal syndrome caused by *TRIM8* variant. It also reminds us to pay attention to eye damage in addition to kidney and nervous system damage.

## Case presentation

**Fig. 1:**
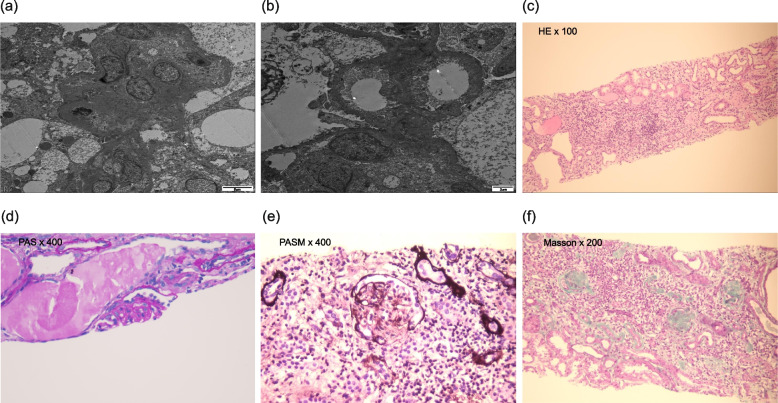
Pathological results of the renal biopsy ((**a**, **b**) Images obtained using electron microscope, (**c**-**f**) Images obtained using light microscope)

A 6-year-old boy presented to our hospital because of urinary incontinence with urgent urination for 3 years. He had astigmatism and myopia. He had no history of convulsions. His physical growth, intelligence, and motor development were normal. There was no family history of consanguineous marriage, kidney disease, or hereditary disease. The physical examination revealed specific facial features, including low-set ears, micrognathia, thin lips, wide eye distance, stubby neck, and large ear lobes. His blood pressure was normal. There was no edema in his body. All other results of the physical examination were normal. The urine examination showed massive proteinuria at the nephrotic level. The blood examination indicated that he had hyperlipidemia, that the lowest level of his serum albumin was 25.3 g/L, and that his estimated glomerular filtration rate suggested stage 3 chronic kidney disease. Urinary ultrasound suggested reduced volume and hydronephrosis in both kidneys. The urodynamic examination showed decreased bladder compliance and poor detrusor contraction. Brain magnetic resonance imaging showed that the bilateral frontal extracerebral space was widened, the left ventricle was fuller than the right ventricle, and a cystoid cerebrospinal fluid signal was observed in the cisterna magna and was considered to be an arachnoid cyst. The Wechsler Intelligence Scale result was normal. After excluding tuberculosis, hepatitis B, systemic lupus erythematosus, diabetes, tumors, and other diseases, he was administered sufficient prednisone, captopril, dipyridamole, and multivitamins orally for 28 days. There was no remission in the proteinuria and serum creatinine levels, but the urinary incontinence symptoms disappeared after treatment with Solifenacin. A further renal biopsy showed diffuse fusion of the foot process and microvilli, but no significant thickening of the glomerular basement membrane was observed using electron microscopy. Using light microscopy, eight glomeruli were observed; of these, six had glomerular sclerosis and one was an incomplete glomerulus with suspected segmental sclerosis. The renal tubules showed cystic dilatation. No capillary endothelial cell proliferation, mesangial cell proliferation, inflammatory infiltration, necrotizing lesions, crescent, or thrombotic microangiopathy was observed. These pathological changes suggested FSGS with sclerosis and cystic dilatation of renal tubules (Fig. 1).

**Fig. 2:**
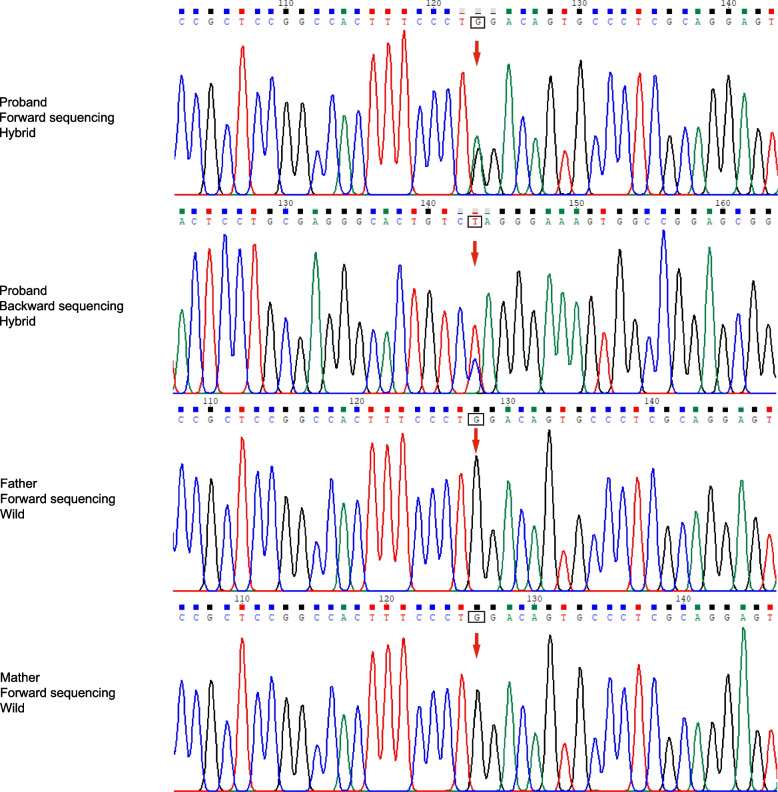
Sanger sequence of the patient and his parents

We performed whole exome sequencing and found a novel de novo truncating variant in the last exon of *TRIM8* (Chr10:104,416,939;NM_030912.2:c.1484G > A;p.Trp495*). This variant leads to the early termination of protein translation encoded by *TRIM8*. There were no additional kidney or nervous-related pathogenic gene variations that could be found in the proband. According to the criteria for classifying pathogenic variants in the guide of sequence variation [12], the variant was classified as pathogenic. Sanger sequencing showed that the variant and other variants were not detected in his parents, suggesting that the variation was a de novo variant in the patient himself (Fig. 2).

## Discussion and conclusions

The pathogenesis of a *TRIM8* variant leading to neurological and renal system diseases is still unclear. The TRIM8 protein is an E3 ubiquitin ligase widely involved in JAK-STAT, NF-κB, γ-interferon, and other signaling pathways [2, 7, 13–15]. The TRIM8 protein normally localizes to the nuclear bodies of renal podocytes and neuronal cells, whereas the *TRIM8* gene variant causes TRIM8 protein to be mislocalized to the nucleoplasm, resulting in protein truncation clustering. Therefore, abnormal nuclear localization may cause the early termination of TRIM8 protein translation, thus interfering with the normal transmission of the aforementioned cellular signal pathways [4, 16–18].

Among the previously reported 22 patients with nervous and renal system diseases caused by the *TRIM8* variant, 17 patients (77%) had both nervous and renal system diseases, three patients (14%) had only nervous system diseases, and two patients (9%) had only renal system diseases. The average age at the time of onset for patients with nervous system involvement is 2.4 years, and most of them present with epilepsy and psychomotor retardation. The average age at the time of onset for patients with renal system involvement is 4.5 years. Renal diseases occur at the same time as or after nervous system diseases. During the early stage, most patients have asymptomatic proteinuria; later, they have nephrotic-range proteinuria. Only a few patients have typical nephrotic syndrome during the early stage. Most patients with renal diseases eventually experience progression to end-stage renal disease. Renal biopsies were performed for 17 of the 22 previously reported patients; two had mesangial glomerulonephritis, two had diffuse mesangial sclerosis, and 13 had FSGS. Of all the patients who underwent renal biopsy, only one case was associated with renal tubular damage (An additional table file shows this in more detail (see Additional file 1)). Weng et al. performed a gene sequencing analysis of 2501 patients with steroid-resistant nephrotic syndrome, 9057 patients with epilepsy, and 48,556 control subjects, and detected *TRIM8* variants in 12 patients (one was previously reported by Warren [7]). They confirmed that *TRIM8* is a single pathogenic gene for renal disease in these patients, and almost all of these patients presented with epilepsy and psychomotor retardation; therefore, it was called neuro-renal syndrome.

**Fig. 3:**
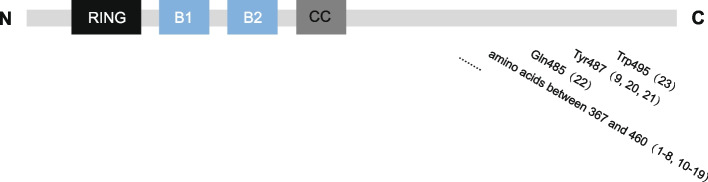
The sites of protein truncation caused by *TRIM8* variants (numbers in parentheses correspond to case numbers in Additional file 1)

We reported a patient with nephrotic proteinuria, specific facial features, and neurogenic bladder, and the whole exon sequencing indicated *TRIM8* variant. Specific facial features, including low-set ears, large ear lobes, micrognathia, and thin lips have been observed in previous cases. Only one patient with enuresis has been reported, but the urodynamic examination was not performed. Our patient had decreased bladder compliance, poor detrusor contraction, and increased residual urine volume in the bladder. Symptoms of the neurogenic bladder were relieved after treatment with Solifenacin. We consider that the neurogenic bladder may be related to the slight involvement of the nervous system caused by *TRIM8* variant. However, he had no seizures or no obvious physical, intellectual, or motor development abnormalities. This symptomatology is consistent with the patients reported by Shirai (case 9 in Additional file 1) and Li (case 22 in Additional file 1) who did not have epilepsy and psychomotor developmental abnormalities, and had only renal disorders as the prominent manifestation [5, 11]. Patients reported by Weng (case 20 in Additional file 1) and Li (case 21 in Additional file 1) had nephrotic-range proteinuria with mild psychomotor retardation, but no seizures [4, 11]. We found that the five patients with no severe neurological symptoms reported by Shirai, Li, Weng, as well as the current case, all shared one common feature: their *TRIM8* variant sites were closer to the C-terminal and all the protein truncation sites were more than 480. Regarding the remaining 18 patients, the protein truncation sites were between 367 and 460 (Fig. 3). Additionally, the variant site of the present patient is closest to the C-terminal, and this has not been reported previously. Therefore, we inferred that when the *TRIM8* variants are closer to the C-terminal, the renal lesions are more prominent and there are fewer neurologic lesions. The pathological changes caused by *TRIM8* variants may not show the typical triad of neuro-renal syndrome (epilepsy, psychomotor retardation, and nephrotic-range proteinuria), and they may be characterized only by renal or nervous system diseases. The reason may be related to the different sites of protein truncation caused by the aforementioned *TRIM8* variants. However, the specific molecular mechanism of this genotype–phenotype correlation requires further study.

Interestingly, no previous studies found a relationship between *TRIM8* variants and ocular lesions. Our patient had myopia and astigmatism. McClatchey reported one patient with strabismus, and Weng et al. reported one patient with hyperopia and astigmatism and one patient with amblyopia and astigmatism [4, 6]. This suggests that, in addition to renal and nervous system diseases, attention should be focused on ocular lesions of patients with a *TRIM8* variant. The lesions may be related to the highest expression of *TRIM8* in the central nervous tissue, kidney, and eye.

In conclusion, we reported a patient with a de novo truncating variant of *TRIM8* with a variant site closest to the C-terminal and nephrotic-range proteinuria but without epilepsy and psychomotor retardation. Additionally, unlike most of the previously reported cases, the renal biopsy did not show renal tubule damage. However, our patient had FSGS with cystic dilatation of the renal tubules. We also reviewed the literature reporting the *TRIM8* variant causing neurological and renal diseases and found an interesting phenomenon: the closer the *TRIM8* variant is to the C-terminal, the more likely it is to cause renal diseases; however, the neurological diseases will not be obvious. Our study provides a new understanding of neuro-renal syndrome caused by *TRIM8* variant. However, this must be confirmed by accumulating more cases and further genetic and molecular studies.

## Supplementary Information

**Additional file 1.** Clinical characteristics of the 22 previous cases and present case with *TRIM8* variant. An additional table file shows more details of the cases with *TRIM8* variant. 

## Data Availability

All data generated during this study are included in this published article.

## Abbreviation

FSGS: Focal segmental glomerulosclerosis
