# Supplementary material for: De novo truncating variants of TRIM8 and atypical neuro-renal syndrome: a case report and literature review
Source: Ital J Pediatr. 2023 Apr 15;49:46. doi: 10.1186/s13052-023-01453-4 (PMC10105407; doi:10.1186/s13052-023-01453-4)
Supplement: Supplementary file 1 — Additional file 1. Clinical characteristics of the 22 previous cases and present case with TRIM8 variant. An additional table file shows more details of the cases with TRIM8 variant. [file 13052_2023_1453_MOESM1_ESM.pdf]

**Additional file 1. Clinical characteristics of the 22 previous cases and present case with *TRIM8* variant**

| No. | Sex | Epilepsy | Age 1[a] | DD[b] | Facial features | Brain magnetic resonance imaging | Others | Renal disease | Age 2[c] | Renal biopsy | Renal outcome | *TRIM8* variants | Ref |
|---|---|---|---|---|---|---|---|---|---|---|---|---|---|
| 1 | M[d] | Yes | 0.4 | Yes | Mild facial features, long philtrum | Widespread moderate cortical atrophy, scattered T2 signal increase in the white matter | Hypospadias | None | NA | NA[e] | NA | p.Ala374Argfs*16 | (10) |
| 2 | M | Yes | 1.8 | Yes | synophridia, sunken eyes, long philtrum | NM[f] | None | None | NA | NA | NA | p.Gln423* | (9) |
| 3 | M | Yes | 0.2 | Yes | Microcephaly, upper-slanted palpebral fissures, long-arched eyebrows, thin lips, large ear lobes | Normal | Hip dislocation, enuresis, constipation | None | NA | NA | NA | p.Cys367Trpfs*43 | (8) |
| 4 | F[g] | Yes | 3.4 | Yes | None | Patchy confluent regions of T2 hyperintensity with small cysts in the frontal and peritrigonal white matter on both sides | Cheerful disposition | Nephrotic-range proteinuria | NM | None | NM | p.Ser444* | (9) |

| | | | | | | | | | | | | | |
|---|---|---|---|---|---|---|---|---|---|---|---|---|---|
| 5 | F | Yes | 1.8 | Yes | Microcephalic, left simian crease, fine hair | Normal brain structures with hypomyelination at the anterior limb of the bilateral internal capsule and bifrontal lobes of the white matter | None | Proteinuria | 0.7 | None | Spontane-ously resolved | p.Gln459* | (9) |
| 6 | F | Yes | 2 | Yes | Gingival overgrowth, synophrys, long philtrum | Delayed myelination | Long extremities, joint hyperlaxity | Nephrotic-range proteinuria | 4 | Mesangial glomerulonephri-tis with nonspecific IgM deposits | NM | p.Tyr446* | (9) |
| 7 | M | Yes | 2.4 | Yes | NM | Normal | None | Nephrotic-range proteinuria | 2.4 | FSGS | ESRD | p.Tyr460* | (7) |
| 8 | M | Yes | 5 | Yes | NM | Identified an old infarct, other brain structures appeared normal | Right-side squint, chronic constipation | Nephrotic-range proteinuria | 2.2 | FSGS | ESRD | p.Tyr400ArgfsTer2 | (6) |
| 9 | F | No | NA | No | None | Normal | None | nephrotic-range proteinuria | 3 | FSGS; cystic dilatation of distal tubules | ESRD | p.Tyr487* | (5) |

| | | | | | | | | | | | | | |
|---|---|---|---|---|---|---|---|---|---|---|---|---|---|
| 10-19 | 4M 6F | Yes | 1.5 to 4.5 | Yes | Broad nasal bridge, broad forehead, plagiocephaly, low-set ears, high arched palate, micrognathia | Except for 1 normal case, the others showed different degrees of abnormality | Increased bladder pressure, hyperopia, astigmatism, amblyopia, sensorineural hearing loss, scoliosis | 8 cases of nephrotic syndrome, 2 cases of nephrotic-range proteinuria | Birth to 13.7 | Two cases of diffuse mesangial sclerosis, the others are FSGS | ESRD | p.Phe388Serfs*2, p.Gly401*, p.Gln411*, p.Gln414*, p.Gln423*, p.Gln445*, p.Tyr460*, Three cases are p.Gln459* | (4) |
| 20 (twins) | M | No | NA | Yes (mild) | None | NM | Both hypotonia, Tourette's syndrome, preserved verbal intelligence quotient, difficulties with fine movements. | Both had nephrotic-range proteinuria | 6 | FSGS | ESRD | p.Tyr487* | (4) |
| 21 | F | No | NA | Yes (mild) | None | Normal | 2 convulsions associated with hypertension (electroencephalogr-am results were normal) | Nephrotic-range proteinuria | 3.1 | FSGS | ESRD | p.Tyr487 | (11) |
| 22 | F | No | NA | No | None | NM | None | Nephrotic-range proteinuria | 3.3 | Glomerular-segmental mesangial | Proteinuria decreased after | p.Gln485 | (11) |

| | | | | | | | | | | | | | |
|---|---|---|---|---|---|---|---|---|---|---|---|---|---|
| | | | | | | | | | | proliferative lesions | treatment | | |
| 23 | M | No | NA | No | Low-set ears, micrognathia, thin lips, wide eye distance, stubby neck, large ear lobes | Slightly wider bilateral frontal extracerebral space, the left lateral ventricle is slightly fuller than the right lateral ventricle, and presence of an arachnoid cyst or cisterna magna | Reduced kidney size, hyperlipidemia, neurogenic bladder, astigmatism, myopia | Nephrotic-range proteinuria | 6 | FSGS; cystic dilatation of renal tubules | Stage 3 chronic kidney disease | p.Trp495* | Present case |

[a]Age 1: age at the time of onset of epilepsy (year)

[b]DD: developmental delay

[c]Age 2: age at the time of onset of renal disease (year)

[d]M: male

[e]NA: not applicable

[f]NM: not mentioned

[g]F: female
